# Intraoperative circulatory arrest secondary to high-risk pulmonary embolism. Case series and updated literature review

**DOI:** 10.1186/s12871-023-02370-z

**Published:** 2023-12-18

**Authors:** Gustavo Cruz, Santiago Pedroza, Miller Giraldo, Alvaro D. Peña, Camilo A. Calderón, Ivan F. Quintero

**Affiliations:** 1 Departamento de anestesiología, Fundación Valle del Lili, Cra 98 No. 18–49, Cali, 760032 Colombia; 2 Centro de investigaciones clínicas, Fundación Valle del Lili, Cra 98 No. 18–49, Cali, 760032 Colombia; 3 Departamento de cardiología y hemodinamia, Fundación Valle del Lili, Cra 98 No. 18–49, Cali, 760032 Colombia; 4 Departamento de cirugía cardiovascular, Fundación Valle del Lili, Cra 98 No. 18–49, Cali, 760032 Colombia; 5 Departamento de cardiología, Fundación Valle del Lili, Cra 98 No. 18–49, Cali, 760032 Colombia

**Keywords:** Cardiac Arrest, Pulmonary Embolism, Intraoperative, High-risk, Thrombolysis. Surgical embolectomy, Anticoagulation

## Abstract

**Background:**

Intraoperative pulmonary embolism (PE) with cardiac arrest (CA) represents a critical and potentially fatal condition. Available treatments include systemic thrombolysis, catheter-based thrombus fragmentation or aspiration, and surgical embolectomy. However, limited studies are focused on the optimal treatment choice for this critical condition. We present a case series and an updated review of the management of intraoperative CA secondary to PE.

**Methods:**

A retrospective review of patients who developed high-risk intraoperative PE was performed between June 2012 and June 2022. For the updated review, a literature search on PubMed and Scopus was conducted which resulted in the inclusion of a total of 46 articles.

**Results:**

A total of 196 174 major non-cardiac surgeries were performed between 2012 and 2022. Eight cases of intraoperative CA secondary to high-risk PE were identified. We found a mortality rate of 75%. Anticoagulation therapy was administered to one patient (12.5%), while two patients (25%) underwent thrombolysis, and one case (12.5%) underwent mechanical thrombectomy combined with thrombus aspiration. Based on the literature review and our 10-year experience, we propose an algorithm for the management of intraoperative CA caused by PE.

**Conclusion:**

The essential components for adequate management of intraoperative PE with CA include hemodynamic support, cardiopulmonary resuscitation, and the implementation of a primary perfusion intervention. The prompt identification of the criteria for each specific treatment modality, guided by the individual patient’s characteristics, is necessary for an optimal approach.

## Backround

Sudden cardiac arrest (CA) is the third leading cause of death in industrialized countries [1]. Within this context, acute pulmonary thromboembolism (PE) has been identified as the confirmed cause in at least 2–5% of cases, although its actual incidence may be much higher due to frequent clinical underdiagnosis [1]. PE is a serious condition caused by partial or complete obstruction of the pulmonary artery or its branches [2]. Its incidence varies between 60 and 200 cases per 100,000 individuals per year, and up to 95% of thrombi originate from the deep venous system of the lower limbs [3]. When PE presents with hemodynamic instability or CA, it carries a high risk of early mortality, ranging from 22 to 32%, reaching up to 65–95% if CA occurs [3–5].

Patients undergoing surgical procedures face an elevated risk of developing deep vein thrombosis (DVT), which can subsequently lead to PE. Surgical procedures can amplify the risk of DVT by up to 70 times for both outpatient and inpatient surgical individuals [6]. This heightened risk isn’t solely due to patient immobility; surgical procedures can induce systemic inflammation, cause vascular injuries, and activate the coagulation system, all of which contribute to thrombotic events [6].

Available treatments for PE include thrombolytic agents, catheter-directed therapies (CDT), and surgical embolectomy (SE) [7]. However, limited studies focus on the optimal treatment of high-risk intraoperative PE associated with CA. We present a case series and an updated review, proposing a flowchart for the diagnosis and adequate treatment of patients with high-risk intraoperative PE associated with CA, highlighting areas that still require clarification and evidence through future research.

## Methods

The study was conducted in accordance with the Declaration of Helsinki and approved by the Institutional Ethics Committee of La Fundación Valle Del Lili in Cali, Colombia (approval number 2023.032, Act No. 12 of 2023). Following the guidelines outlined in resolution 8430 of 1993, this research was deemed to pose no risks. As a result, the exemption from obtaining informed consent was both sought and acquired. The study contained no mention of personal identification in any form.

We performed a retrospective review of 8 patients who developed CA secondary to high-risk intraoperative PE between June 2012 and June 2022 at Fundación Valle Del Lili, Cali, Colombia. Inclusion criteria encompassed the occurrence of CA due to high-risk PE, defined as systolic blood pressure below 90 mmHg for more than 15 min, clinical signs of shock due to inadequate blood flow, and the exclusion of other potential causes. The PE diagnosis was made by transesophageal echocardiography (TEE) and/or computed tomography angiography (CTA).

Data was collected by reviewing patients’ medical records in the SAP system, a technological platform facilitating data recording and integration. We gathered information on patients’ demographic characteristics (such as gender and age), medical history, current diagnosis, and surgical procedures. The preoperative risk of deep vein thrombosis for each patient was calculated using the CAPRINI scale. Outcome measures included the initial clinical presentation of PE, CA characteristics, and mortality. High vasopressor support treatment was defined as a norepinephrine dose > 0.2ug/kg/min and vasopressin > 0.04 UI/min. Additionally, we documented treatment modalities, including thrombolysis, mechanical thrombectomy, SE, and extracorporeal membrane oxygenation (ECMO).

To conduct the updated review, a literature search in PubMed and Scopus was performed. The mesh terms “pulmonary embolism” AND “cardiac arrest” AND “Intraoperative” were used. Additional terms were included to enhance the search, such as “anticoagulation,” OR “thrombolysis,” OR “surgical embolectomy” OR “catheter-directed thrombolysis” OR “mechanical thrombectomy.”

**Fig. 1:**
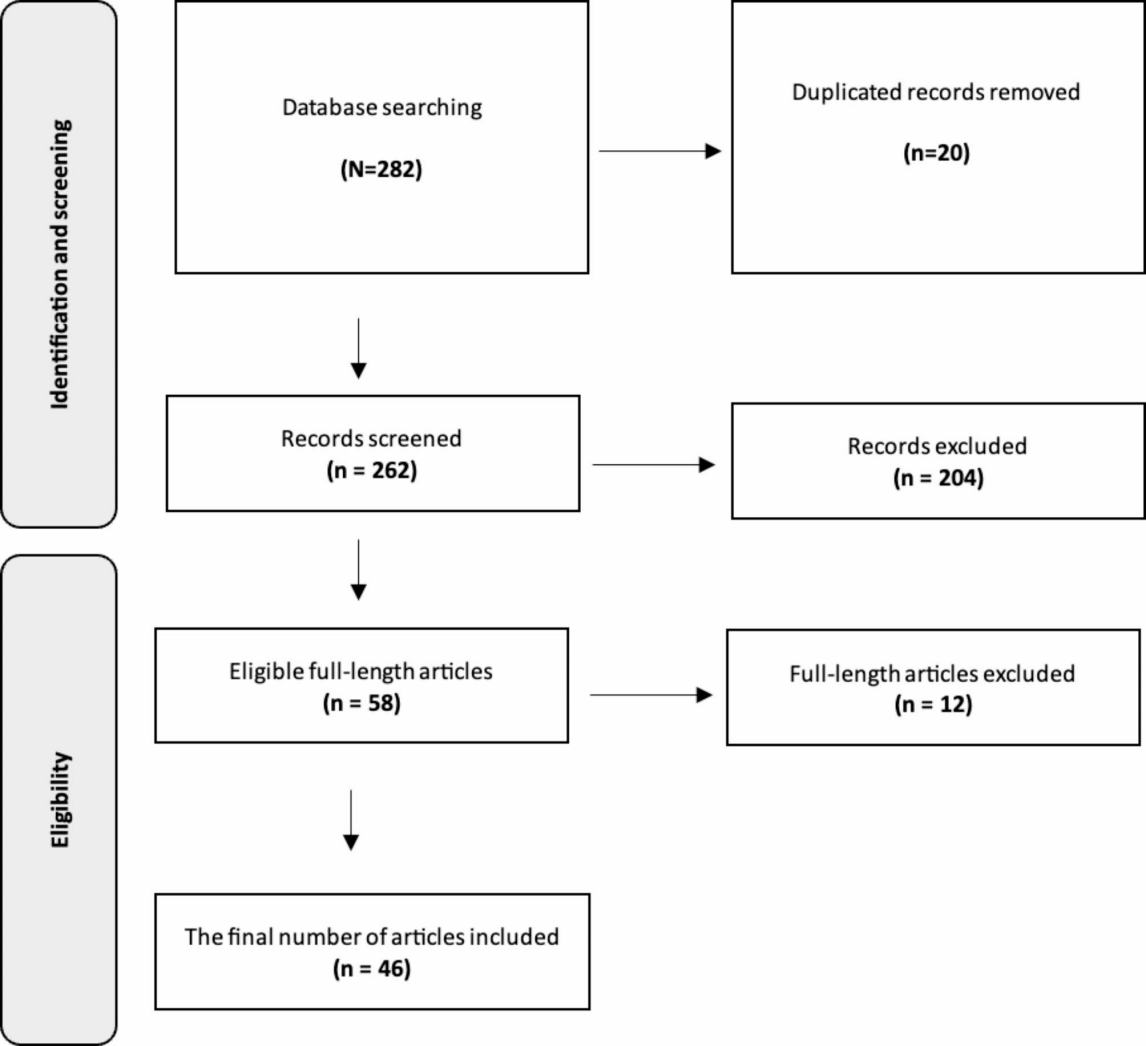
Process of article selection

A total of 58 articles were eligible for full-length review. The selection of the final articles was based on the researchers’ criteria, resulting in the inclusion of 46 articles (Fig. 1). We employed the 2020 PRISMA guidelines for systematic review reporting.

A total of 58 articles were eligible for full-length review. The selection of the final articles was based on the researchers’ criteria, resulting in the inclusion of 46 articles (Fig. 1). We employed the 2020 PRISMA guidelines for systematic review reporting. Twelve full-length articles were omitted from our review due to three primary considerations: the lack of direct relevance to the intraoperative management of high-risk pulmonary embolism, insufficient data on outcomes following cardiac arrest, or methodological limitations that could potentially bias the results.

## Results

A total of 196 174 major non-cardiac surgeries were performed between 2012 and 2022. Eight cases of CA due to high-risk intraoperative PE were identified. The average age of the patients was 64 (SD: 22), with 75% being female. Arterial hypertension was the most prevalent associated medical condition (63%). Four patients had a history of previous PE, and one patient had a diagnosis of obesity. Among the procedures, 63% were classified as urgent, including major orthopedic and abdominal surgeries. The elective procedures consisted of major orthopedic surgeries and one case of transurethral resection of the prostate (TURP). The risk of thrombotic events, assessed by the CAPRINI scale, was categorized as moderate in 3 patients (37.5%) and high in 5 patients (62.5%) (Table 1).

**Table 1:** Patient characteristics, medical history, and risk factors

Variables	N = 8	% or mean ± SD
**Age**	64 ± 22 27.6 ± 3.7	
**BMI**		
**Sex**		
Female	6	75.0%
Male	2	25.0%
**Concomitant diseases/RF**		
HTN	5	62.5%
Previous PE	4	50.0%
Hip/knee fracture	3	37.5%
PH	2	25.0%
Hypothyroidism	2	25.0%
Malignity	2	25.0%
APS	2	25.0%
DVT	1	12.5%
Diabetes	1	12.5%
Obesity	1	12.5%
History of smoking	1	12.5%
Parkinson	1	12.5%
CKD	1	12.5%
SLE	1	12.5%
AAA	1	12.5%
Urgent surgery	5	62.5%
Pregnant	1	12.5%
Limited mobility	5	62.5%
Recent surgeries	3	37.5%
**Caprini score (RISK)**		
Low	0	0.0%
Medium	3	37.5%
High	5	62.5%

SD: Standard deviation, BMI: Body Mass Index, RF: risk factors, HTN: Hypertension, PE: Pulmonary embolism, PH: Pulmonary hypertension, APS: Antiphospholipid syndrome, DVT: Deep vein thrombosis, CKD: Chronic kidney disease, SLE: Systemic lupus erythematosus, AAA: Abdominal aortic aneurysm

In terms of CA presentation, 6 patients (75%) experienced at least one episode of pulseless electrical activity (PEA), while 4 patients (50%) had at least one episode of asystole. Advanced cardiopulmonary resuscitation (CPR) was performed in all cases, with variations in medication use (such as atropine, adrenaline, or other vasoactive agents) based on individual circumstances. Anticoagulation therapy was administered to one patient (12.5%), while two patients (25%) underwent thrombolysis, and one case (12.5%) underwent mechanical thrombectomy (MT) combined with thrombus aspiration. A SE was not performed in any of the cases. The mortality rate was 100% for patients who experienced asystole, in contrast with 50% for those who only had PEA. The overall mortality rate was 75%.

TEE performed by the cardiovascular anesthesia team confirmed the diagnosis of PE in all patients during CA. The diagnosis was established based on the visualization of a mobile thrombus within the right cardiac chambers or in the pulmonary trunk and its branches. Alternatively, it was made due to the sudden dysfunction of the right ventricle, accompanied by severe tricuspid insufficiency and a reduction in left ventricular preload, which could not be explained by other conditions. Only one patient with low vasopressor support underwent CTA to guide specific therapy for PE.

## Discussion

High-risk PE is a severe condition, characterized by hemodynamic instability and right ventricular (RV) dysfunction [8]. In our case series, we obtained a mortality rate of 75% in patients with CA due to high-risk PE, similar to the rates reported in the literature (65–95%) [3–5]. Given the high mortality of this condition, early diagnosis, risk stratification, and implementation of aggressive therapeutic strategies are essential [9].

### Cardiac arrest rhythm

Limited research exists on the difference in mortality based on the CA rhythm. However, evidence suggests that PEA is associated with a lower mortality rate compared to asystole. In a study involving 14,720 patients with CA, the survival rate was reported as 35% for asystole and 39% for PEA [10]. Similarly, Høybye et al. demonstrated a higher survival rate for PEA compared to asystole [11].

Our study revealed a higher survival rate (50%) among patients who experienced only PEA episodes, in contrast to a 0% survival rate in patients with asystole episodes. Additionally, all patients with PEA episodes achieved a ROSC following initial resuscitation, regardless of the outcome. Consequently, the presence of asystole is considered a high-risk indicator, warranting the consideration of immediate thrombolysis or second-line interventions such as ECMO in conjunction with SE or CDT. Nevertheless, further investigations are necessary to validate this recommendation.

### Initial treatment

When identifying a patient with high-risk PE and CA, it is crucial to ensure hemodynamic support and provide specific treatment for PE. Various interventions are available to achieve hemodynamic support, including intravenous fluid administration, vasopressors, inhaled nitric oxide, positive pressure mechanical ventilation (PPMV) with minimal positive end-expiratory pressure, and, in some cases, ECMO [4].

#### Ventilation

It is important to consider the potential adverse hemodynamic effects of PPMV, particularly the reduction in venous return and exacerbation of low cardiac output due to positive intrathoracic pressure. The 2019 ESC guidelines recommend ventilation volumes of approximately 6 mL/kg of lean body weight to maintain the inspiratory plateau pressure below 30 cm H2O [12]. Some case reports and small clinical studies suggest that inhaled nitric oxide may improve gas exchange and hemodynamic status in high-risk PE patients [12–14], however, conclusive clinical evidence regarding its efficacy and safety is currently lacking [12].

#### Fluid therapy

In terms of intravenous fluid therapy, a modest initial fluid load of less than 500 mL over 15–30 min is recommended for patients with low central venous pressure, as it can enhance cardiac index. However, it should be noted that volume overload can lead to overdistension of the right ventricle and a subsequent decrease in cardiac output. Experimental studies suggest that aggressive volume expansion offers no benefit and may even worsen RV function [8, 12].

#### Vasopressors

Vasopressors are needed alongside reperfusion treatment in high-risk PE with CA. Norepinephrine, for instance, can improve ventricular systolic function and coronary perfusion without significantly affecting pulmonary vascular resistance. However, its administration should be limited to patients in cardiogenic shock, with a recommended dose range of 0.2-1.0 µg/kg/min (Class IIa, Level C evidence) [12].

#### ECMO

In cases of RV failure following a CA that is unresponsive to initial hemodynamic support or in the presence of refractory circulatory collapse, ECMO is recommended in combination with SE or CDT (Class IIb, Level C evidence) [4, 12]. Furthermore, ECMO should be considered for patients with potential contraindications to thrombolysis. ECMO maintains systemic perfusion while relieving the right ventricle, rendering it an effective therapeutic approach for CA due to PE. Although several studies have shown promising results with this approach, it is important to acknowledge their limitations, including small sample sizes and potential selection biases [15].

### Diagnosis

The diagnosis of intraoperative PE in CA and anesthetized patients presents a considerable challenge due to the atypical presentation of signs and symptoms. Unexplained hypoxia, tachycardia, hypotension, and/or decreased levels of expired end-tidal carbon dioxide should alert healthcare providers to the possibility of PE. Consequently, prompt, and thorough diagnostic evaluation becomes essential.

The collaborative consensus statement by the American Society of Echocardiography and the Society of Cardiovascular Anesthesiologists recommends TEE in scenarios characterized by persistent, life-threatening circulatory instability, which remains unexplained despite appropriate therapeutic interventions (Class I, Level C evidence) [12, 16]. Moreover, studies have provided evidence of comparable levels of sensitivity and specificity between TEE and computed tomography in the evaluation of centrally located PE [17].

TTE can reveal hypokinesis and RV dysfunction, suggesting high-risk PE. A study from the International Cooperative Pulmonary Embolism Registry demonstrated that RV hypokinesis on TTE was associated with a twofold increased risk of mortality in patients with PE [18]. Therefore, in unstable patients, evidence of RV dysfunction on TTE is sufficient to prompt immediate primary pulmonary reperfusion without the need for additional tests [19]. Pulmonary CTA should be performed in stabilized patients (low vasopressor support) to confirm the diagnosis and guide specific PE treatment (Class I, Level C evidence) [3, 12].

### Anticoagulation

Anticoagulation is the cornerstone of treatment for PE [20]. It should be initiated immediately upon diagnosis of PE or in the presence of high clinical suspicion (Class I, Level C evidence) [3, 12]. However, in patients with CA and high-risk PE, treatment escalation is warranted [21]. According to the European Resuscitation Council (ERC) recommendations, thrombolysis, SE, and CDT should be considered as the primary reperfusion treatment options in these cases [12]. Anticoagulation should be considered a viable primary perfusion treatment in patients with low vasopressor support after a complete ROSC, with no indications of other primary perfusion therapies.

During surgical procedures, using unfractionated heparin (UFH) may be preferable due to its shorter half-life, dose adjustability, and potential reversal with protamine [22]. The 2019 ESC guidelines for the management of PE recommend the use of UFH in patients with evident hemodynamic instability or decompensation who require primary reperfusion treatment [12]. UFH is also recommended for patients with severe renal insufficiency (< 30 mL/min) or severe obesity [12].

### Systemic thrombolysis

Traditionally, major surgery and the diagnosis of CA have been considered contraindications for systemic thrombolysis [23]. However, the concept of administering thrombolytic agents during these situations associated with high-risk PE is increasingly supported by case reports and clinical studies.

Tissue plasminogen activator (tPA) is the most studied thrombolytic agent and has a greater number of approved indications, making it the recommended thrombolytic choice (Class I, Level B evidence) [12]. Regarding systemic thrombolysis in CA and high-risk PE, the most recent study, conducted by Javaudin et al., highlights the benefits of this therapeutic approach in terms of overall survival, with a clear trend toward reducing mortality [24]. This is one of the strongest pieces of evidence regarding the concept of thrombolysis during CPR in PE, as it is questionable whether any randomized controlled trial would be feasible or ethical for this condition.

Similarly, Kurkciyan et al. assessed the effect of tPA in patients with CA suspected of PE. 63% (83/132) of patients in the tPA group survived compared to 35% (47/133; *P* < 0.001) in the control group [23]. These studies, along with other case reports [5, 9, 15, 19], support the notion that CPR is not an absolute contraindication for systemic thrombolysis. Additionally, both the ERC and the American Heart Association (AHA) have recommended the use of fibrinolytic therapy when PE is suspected or known to be the cause of CA [12, 21]. Once administered, CPR should continue for at least 60–90 min (successful cases with over 100 min of CPR have been reported).

Moreover, the decision for intraoperative thrombolysis as a rescue treatment for high-risk PE can be challenging due to the substantial risk of massive hemorrhage. However, the potential survival benefit of fibrinolysis outweighs the potential risks if no other alternative is available [1]. Thirteen case reports were found in which intraoperative systemic thrombolysis was used for high-risk PE (Table 2).

**Table 2:** Case reports of intraoperative systemic thrombolysis for high-risk PE

Article type	Author	Year	Surgery	Thrombolytic	Outcome
5 case series [25]	L. Scheeren	1994	Surgical VT	tPA: 20 to 90 mg	3 survivals, NNS 2 deaths
Case report [26]	D. Jackson	2006	Liver Transplant	tPA: two 50 mg doses	Survived, NNS
Case report [27]	M. Wenk	2011	Cesarean section	Reteplase: two 10 UI doses	Survival, NNS
2 Case series [28]	S. Aniskevich	2015	Liver Transplant	tPA: 50 mg divided in 10-mg bolus followed by 5-mg increments every 5 min	1 Death 1 Survival, lower extremity weakness
Case report [29]	J. Cao	2015	Distal femur fracture surgery	tPA: 5 mg initial dose followed by infusion of 45 mg/h	Survival, NNS
Case report [30]	K. Roy	2018	Coronary artery bypass graft	tPA: 10 mg initial bolus followed by infusion of 90 mg/h	Survival, NNS
Case report [31]	R. Holland	2020	Spine surgery	tPA: two 50-mg doses.	Survival, NNS
Case report [32]	A. Karakosta	2023	Cesarean Section	tPA: 15 mg	Survival, NNS

VT: Venous thrombectomy, tPA: alteplase, NNS: no neurological sequelae

Massive hemorrhage occurred in all cases. However, 10 survivors out of 13 cases were reported, and after hemorrhage control and resuscitation, only one patient had neurologic impairment consisting of lower extremity weakness. Additionally, the dosage of tPA varied throughout the literature, ranging from 5 to 100 mg as an initial bolus. Forms of administration included bolus, infusion, or a combination of both (Table 2). From the literature available, it seems that the majority of surgeries could be subjected to thrombolysis, despite contraindications.

The risk of major bleeding has prompted professionals to explore alternative fibrinolysis strategies with lower bleeding risk. Doses of 0.6 mg/kg of tPA have demonstrated effectiveness in preventing mortality in high-risk PE [33, 34]. Furthermore, a case of PE associated with CA in a patient with cirrhosis and thrombocytopenia (high risk of bleeding) was reported, in which a total dose of 12 mg of tPA was administered, resulting in symptomatic improvement and no evidence of residual thrombus on follow-up ETT [35]. Similarly, doses up to 2 mg of tPA have been reported with satisfactory results [34].

Further successful cases of systemic thrombolysis in absolute contraindications have been documented. For instance, Reisinger et al. achieved successful treatment of a high-risk PE in a patient with a history of significant intracranial hemorrhage [36]. Likewise, Koroneos reported a successful case of thrombolysis in a patient experiencing CA associated with PE, despite a previous intracranial hemorrhage [37]. Other reports with contraindications include intracranial neoplasms, arteriovenous malformations, and prior cesarean Sect. [36].

These current data suggest the need to reconsider the traditional contraindications for thrombolysis in PE. Individualized decision-making and a multidisciplinary team focused on the best interest of the patient, is likely the most appropriate approach in complex cases of a similar nature. Furthermore, some of the mentioned case reports suggest that a low dose of tPA could serve as an effective treatment option for intraoperative CA secondary to PE, especially when alternative management options are unavailable. However, further studies are required to determine the optimal dosage of tPA based on the patient’s clinical condition.

### Invasive therapies

Within the realm of invasive therapies, endovascular treatments, and SE are encompassed. Currently, these two therapeutic modalities are recommended for patients with high-risk PE for whom thrombolysis is either contraindicated or has proven unsuccessful. SE is supported by Class I, Level C evidence, while CDT receives a Class IIa, Level C recommendation [19].

#### Surgical embolectomy

SE enables access to the thoracic cavity through a sternotomy, followed by incisions in the two primary pulmonary arteries to extract or aspirate the clots. This surgical approach has undergone significant reconsideration as a treatment option for high-risk PE. Advances in cardiopulmonary bypass surgery and modern surgical techniques have contributed to a noteworthy reduction in associated mortality rates.

This assertion is substantiated by several studies. For instance, Kilic et al. reported up to 27.2% mortality rates in SE performed between 1999 and 2008 [38, 39]. Similarly, Alqahtani et al. documented a mortality rate of 23.1% for SE between 2003 and 2009, while those conducted between 2009 and 2014 displayed a mortality rate of 14% [38, 39]. Additionally, Pasrija et al. observed a 7% mortality rate in a recent retrospective study involving 55 patients [40].

SE is regarded as a definitive therapeutic approach that diminishes the risk of recurrent PE and ensures clot extraction [39]. Furthermore, it serves as an option when thrombolysis fails. A study conducted by Meneveau et al. showed that repeated thrombolysis was associated with a mortality rate of 38%, whereas rescue SE demonstrated a mortality rate of 7% [39, 41]. With time, this therapeutic approach holds the potential to be considered a viable first-line alternative to systemic thrombolysis and anticoagulation. However, further studies comparing surgical therapy with CDT and non-invasive approaches are required to reach more definitive conclusions.

Additional circumstances warrant consideration for surgical thrombectomy (Table 3). These include the echocardiographic identification of an embolus lodged in a patent foramen ovale or in regions proximal to the heart, such as the right ventricle, the main pulmonary artery, and its extrapulmonary branches [18]. Furthermore, an angiographic Miller score > 20/34 supports the use of embolectomy [42]. Thrombi situated in more distal regions, such as the intrapulmonary branches of the pulmonary artery, generally do not qualify for surgical intervention. Lastly, pregnancy constitutes an indication for this treatment modality due to the risk of uterine bleeding. Three case reports have been published on pregnant women who underwent SE, with a 100% survival rate and a fetal/neonatal mortality rate of 25% [43].

**Table 3:** Indications for surgical thrombectomy in high-risk PE patients associated with cardiac arrest

Failure of systemic thrombolysis	
Patent foramen ovale	
Detection of an embolus in the right chambers	
Central emboli	
Miller score > 20/34 (on CTA)	
Pregnancy	
**Contraindication for systemic thrombolysis** - SNC neoplasia - History of intracranial hemorrhage - Stroke (within the last 3 months) - Surgery or major trauma in the past month - Hemorrhagic diathesis - Active bleeding	

CNS: Central nervous system, CTA: computed tomography angiography

Choosing between SE and CDT can be challenging. SE exhibits practical utility in patients necessitating mechanical right ventricular and/or pulmonary support. Moreover, SE shows potential superiority over CDT in individuals with a central PE and a notable embolic burden. In contrast, CDT may present as a more suitable alternative for patients contraindicated for or confronted with substantial risks associated with cardiac surgery, as well as cases with a peripheral clot that is less amenable to surgical intervention [44].

#### Catheter-directed therapy

The available options for endovascular treatment include CDT and MT. CDT offers the advantage of requiring only a fraction of the systemic fibrinolytic dose. This approach can rapidly improve RV function while minimizing the risk of intracranial hemorrhage [16]. MT can be performed through aspiration, fragmentation, or a combination of techniques [17]. The knowledge regarding catheter-based embolectomy largely stems from registries and combined outcomes of case series, reporting a success rate of 87%. However, these results may be influenced by publication bias and the relatively small sample size [45].

Whenever feasible, it is advisable to review CTA images before making decisions about invasive therapy. Evaluating the extent of emboli and the location of clots in the pulmonary arteries assists in appropriately planning the procedure. Most patients with PE and right ventricular dysfunction have emboli located in proximal pulmonary arteries, which are easily accessible for SE. However, patients with clots in more distal or segmental locations pose greater challenges, particularly in less-experienced centers. In such cases, CDT may be a preferable option. The primary limiting factor of these interventions is the clot size [2]. Nevertheless, these emerging minimally invasive techniques have the potential to become first-line treatments, as they provide rapid hemodynamic improvements with low bleeding rates. However, due to the scarcity of studies and limited data, they have not yet become standardized therapies for high-risk pulmonary embolism, unlike thrombolytic agents [2].

It is essential to emphasize that the primary objective of CDT is to achieve hemodynamic stabilization in patients. Hemodynamic stability is considered a clinical success. To achieve this, only a partial reduction of the clot within the pulmonary arteries is necessary. Attempting complete clot removal during a CDT procedure is unnecessary and may entail potential risks, including potential arterial wall damage and increased use of contrast media [46].

### Selection of management strategy

CA secondary to PE should be treated in an aggressive way, with an emphasis on reperfusion therapy. Selecting the most suitable management is a challenge and requires an individualized approach. It relies primarily on the expertise of a multidisciplinary team of specialists rather than solely relying on available evidence. The 2019 ESC guidelines recommend the establishment of a multidisciplinary Pulmonary Embolism Response Team (PERT) to discuss the management of PE cases that carry a high risk [12]. This team should consist of specialists who have practical experience in the acute management of PE, such as anesthesiologists, emergency medicine specialists, cardiology experts, radiologists, hematologists, pulmonologists, and/or, intensive care specialists, depending on the available local resources and circumstances [12].

### Treatment in our institution

In our case series, four patients did not receive specific treatment for PE (Table 4). The reasons were multifaceted, primarily due to the contraindications of the available specific treatments, such as anticoagulation or thrombolysis, in the context of the patient’s major surgeries. The PERT team considered that the magnitude of these surgical procedures presented a substantial risk of massive bleeding, which posed a greater immediate threat to the patient’s survival than the potential benefits of treating the PE. Moreover, the rapid progression of the disease, with a very short interval between the diagnosis of PE and subsequent death, left an insufficient window for the initiation of such treatments.

**Table 4:** Presentation, treatment, and outcome of intraoperative high-risk pulmonary embolism case series

Sex, Age, Date	Surgery	CA Rhythm	CPR* (min)	ROSC	Support*	CTA	ST	Outcome
**F, 83**, **12/11/2014**	HA	Asystole	12	No	High	No	Alteplase 50 mg IV	Dead
**M, 47**, **30/07/2015**	RN	PEA (15 m) Asystole (7 m)	20	No	High	No	No	Dead
**F, 21, 03/10/2015**	Cesarean section	PEA (5 m) ROSC (60 m) PEA (20 m)	20	Yes	High	No	No	Dead
**F, 55**, **31/05/2017**	TH	5 EP PEA	15	Yes	High	No	Alteplase 50 mg IV	Dead
**F, 89**, **17/04/2018**	HA	PEA (7 m) ROSC (20 m) Asystole (15 m)	15	Yes	High	No	No	Dead
**M, 73**, **15/12/2019**	TURP	Asystole (12 m)	12	No	High	No	No	Dead
**F, 68**, **12/07/2022**	BARFC	PEA (3 m)	3	Yes	Low	Yes	Enoxaparin40mg/12H	Survived NNS
**F, 74**, **21/09/2022**	TKR	PEA (3 m) ROSC (25 m) PEA (4 m)	4	Yes	High	No	MT	Survived NNS

CA: cardiac arrest, TEE: Transthoracic Echocardiogram, ROSC: Return of Spontaneous Circulation after resuscitation and hemodynamic support, CTA: Computed Tomography Angiography, ST: Specific Therapy, F: Female, M: Male, EP: Episode, PEA: Pulseless electrical activity, TH: Total Hysterectomy, TURP: Transurethral Resection of the Prostate, HA: Hip arthroplasty, RN: Radical Nephrectomy, MT: Mechanical Thrombectomy, TKR: Total Knee Replacement, BARFC: Bilateral Anterior Rheumatic Forefoot Correction, NNS: no neurological sequelae

*CPR: cardiopulmonary resuscitation of the last episode of cardiac arrest

*High support: norepinephrine > 0.2ug/kg/min and vasopressin > 0.04 UI/min

*Low support: norepinephrine < 0.2ug/kg/min and/or vasopressin < 0.04 UI/min

A single MT (Table 4: F,74) was performed, resulting in complete patient recovery. The PERT team excluded the alternative of surgical embolectomy due to the high surgical risk. Anticoagulation and thrombolysis were contraindicated in this case due to recent orthopedic trauma and major orthopedic surgery involving a total right knee replacement. Additionally, a CT angiography was not performed due to the patient’s hemodynamic instability and high vasopressor support.

MT became available in our institution in 2020. It is plausible to surmise that individuals who underwent thrombolysis, and those who did not receive specific therapeutic interventions (Table 4) due to contraindications related to anticoagulation or thrombolysis, could have potentially achieved more favorable clinical outcomes if afforded the alternative options of mechanical or surgical thrombectomy.

**Fig. 2:**
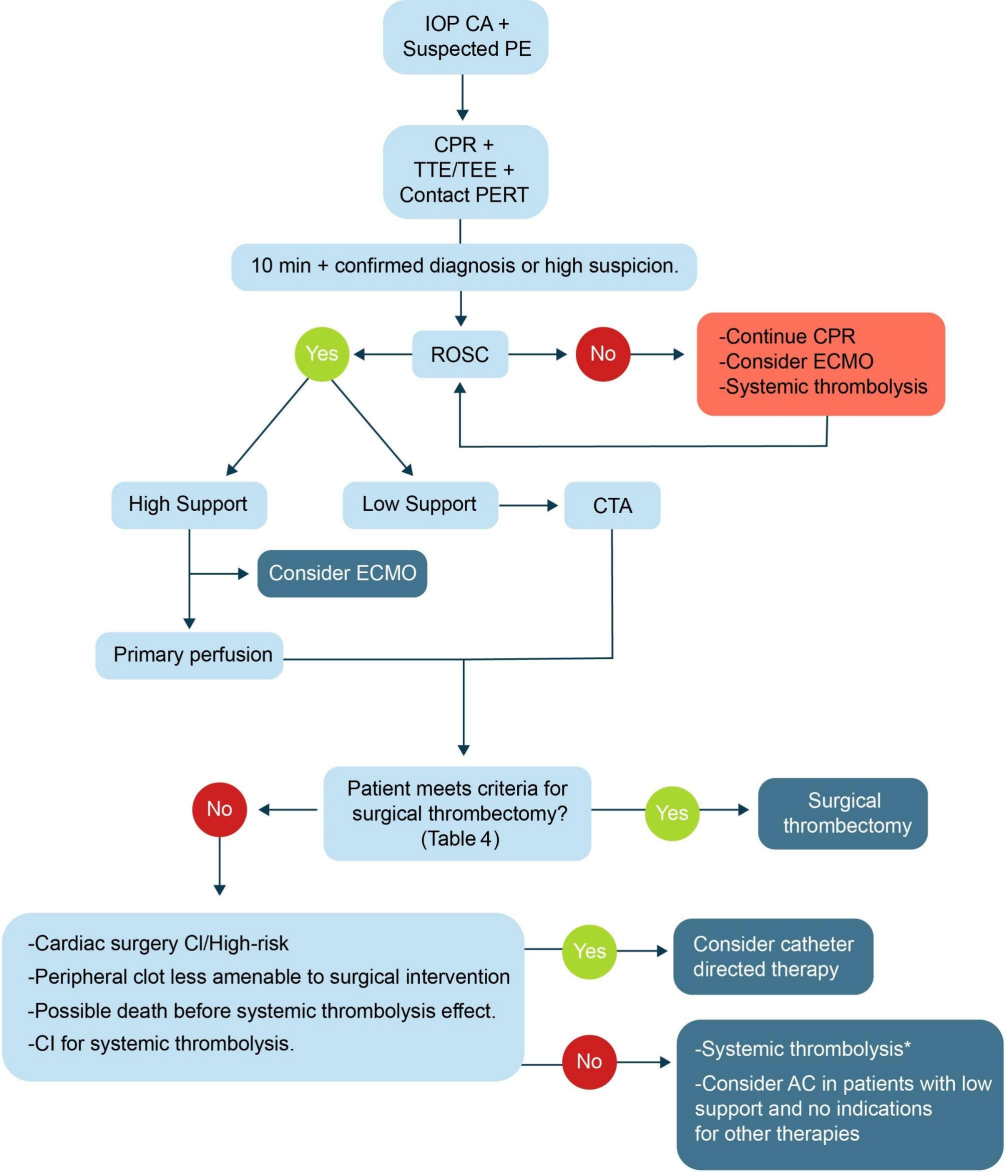
Diagnosis and treatment of intraoperative cardiac arrest with suspected high-risk pulmonary embolism IOP: intraoperative, CA: cardiac arrest, PE: pulmonary embolism, TEE: transthoracic echocardiogram, TEE: transesophageal Echocardiogram, PERT: pulmonary embolism response team, ROSC: Return of Spontaneous Circulation, CPR: cardiopulmonary resuscitation, ECMO: extracorporeal membrane oxygenation, CTA: computed tomography angiography, CI: contraindication, AC: anticoagulation *High support: norepinephrine > 0.2ug/kg/min and vasopressin > 0.04 UI/min *Low support: norepinephrine < 0.2ug/kg/min and/or vasopressin < 0.04 UI/min

Below, we propose a diagnostic and treatment algorithm for patients with CA and suspected high-risk pulmonary embolism, based on the literature review and our 10-year experience (Fig. 2). However, it is important to note that the algorithm for this condition is complex and should be based on established protocols, with the collaboration of multidisciplinary teams, considering the experience and available resources at each institution.

## Conclusions

Intraoperative PE with CA represents a critical and potentially fatal condition. The essential components for delivering prompt and effective management encompass initial hemodynamic support, CPR, accurate determination of PE as the underlying cause of arrest, and implementation of tailored therapeutic interventions. The prompt identification of the criteria for each specific treatment modality, guided by the individual patient’s characteristics, is necessary for an optimal approach. To facilitate this process, we propose an algorithm delineating the diagnostic and specific treatment strategy for PE and CA.

## Data Availability

The datasets used and/or analyzed during the current study are available from the corresponding author upon reasonable request.

## Abbreviations

AHA: American Heart Association
CA: Cardiac arrest
CDT: Catheter-directed therapies
CTA: Computed tomography angiography
CPR: Cardiopulmonary resuscitation
DVT: Deep vein thrombosis
ECMO: Extracorporeal membrane oxygenation
ERC: European Resuscitation Council
MT: Mechanical thrombectomy
PE: Pulmonary embolism
PEA: Pulseless electrical activity
PERT: Pulmonary embolism response team
PPMV: Positive pressure mechanical ventilation
RV: Right ventricular
SE: Surgical embolectomy
TEE: Transesophageal echocardiography
tPA: Tissue plasminogen activator
TURP: Transurethral resection of the prostate
UFH: Unfractionated heparin
